# Photoplethysmography for Quantitative Assessment of Sympathetic Nerve Activity (SNA) During Cold Stress

**DOI:** 10.3389/fphys.2018.01863

**Published:** 2019-01-09

**Authors:** Karthik Budidha, Panayiotis A. Kyriacou

**Affiliations:** Research Centre for Biomedical Engineering (RCBE), School of Mathematics, Computer Science & Engineering, City, University of London, London, United Kingdom

**Keywords:** sympathetic nerve activity, parasympathetic activity, Pulse Transit Time, low-frequency spectral analysis, peripheral circulation, cold stress, vasoconstriction

## Abstract

The differences in the degree of sympathetic nerve activity (SNA) over cutaneous blood vessels, although known to be more prominent in the periphery than the core vasculature, has not been thoroughly investigated quantitatively. Hence, two studies were carried out to investigate the differences in SNA between the periphery and the core during the cold pressor test (CPT) (right-hand immersion in ice water) and cold exposure (whole body exposed to cold air) using photoplethysmography (PPG). Two methods utilizing PPG, namely differential multi-site PTT measurements and low-frequency spectral analysis were explored for quantitative determination of SNA. Each study involved 12 healthy volunteers, and PPG signals were acquired from the right index finger (RIF), left index finger (LIF) (periphery) and the ear canal (core). During CPT, Pulse Transit Time (PTT) was measured to the respective locations and the mean percentage change in PTT during ice immersion at each location was used as an indicator for the extent of SNA. During cold exposure, the low-frequency spectral analysis was performed on the acquired raw PPGs to extract the power of the sympathetic [low-frequency (LF): 0.04–0.15 Hz] and parasympathetic components [high-frequency (HF): 0.15–0.4 Hz]. The ratio of LF/HF components was then used to quantify the differences in the influence of SNA on the peripheral and core circulation. PTT measured from the EC, and the LIF has dropped by 5 and 7%, respectively during ice immersion. The RIF PTT, on the other hand, has dropped significantly (*P* < 0.05) by 12%. During the cold exposure, the LF/HF power ratio at the finger has increased to 86.4 during the cold exposure from 19.2 at the baseline (statistically significant *P* = 0.002). While the ear canal LF/HF ratio has decreased to 1.38 during the cold exposure from 1.62 at baseline (*P* = 0.781). From these observations, it is evident that differential PTT measurements or low-frequency analysis can be used to quantify SNA. The results also demonstrate the effectiveness of the central auto-regulation during both short and long-term stress stimulus as compared to the periphery.

## 1. Introduction

The primary response of the human body to low ambient temperature exposure (cold stress) is the activation of the sympathetic nervous system (SNS) to counter heat loss and maintain homeothermy. The human body maintains homeostasis by a feedback system that includes thermoreceptors, afferent nerve fibers, a hypothalamic control center, efferent nerve fibers, and thermoeffectors (Wehrwein et al., 2016). When exposed to cold, the thermoreceptors in the skin detect the decrease in temperature and send this information to the anterior portion of the hypothalamus that controls the heat balance called the preoptic area, via the afferent nerves. The control center in the preoptic area propagates control signals to other areas in the hypothalamus that regulate the rate of heat production. When behavioral options are unavailable or ineffective for maintaining thermal homeostasis, the heat production center stimulates the efferent nerves in the SNS (Gordon, 2009). This activation leads to a prompt change in cutaneous blood flood through an intense constriction of the skin resistance vessels, the arteriovenous anastomoses (AVA), and the veins in the skin (i.e., thermoeffector). Concurrently, involuntary muscle contraction and a marked increase in heart rate, cardiac output, and blood pressure occur. Through this mechanism, heat transfer from the body's core to the body's surface is attenuated, skin temperature is decreased, and less heat is lost (Stocks et al., 2004).

Once the body is acclimatized to cold, the calming side of the autonomic system i.e., the parasympathetic system, will act as an antidote to the effects of cold stress created by the sympathetic system (Folk, 1981; Kaciuba-Uscilko and Greenleaf, 1989; Daanen and Van Marken Lichtenbelt, 2016). The effects it produces on the body are opposite to sympathetic activity. These effects include an increase of blood flow to the periphery, slowing of the heart rate, and reduced blood pressure. Through these effects, the parasympathetic nerves help the body return to a normal state. The autonomic state resulting from the sympathetic and parasympathetic influences is known as the “sympathovagal balance” (Flavahan, 2015). In normal subjects, the sympathovagal balance fluctuates throughout the day depending on the nature of the activity. During cold stress, the balance shifts more toward the sympathetic activity (raised sympathetic tone). The hemodynamic processes by which the balance is achieved is known as autoregulation (Stocks et al., 2004).

The degree of the sympathetic control over cutaneous blood vessels during cold exposure is, known to be not uniform across the body's vascular system and it is less prominent in the core vasculature compared to the periphery, particularly in the cerebral and facial vasculature. The cerebral vessels deliver a constant supply of blood even when there are wide fluctuations in the systemic blood pressure to limit intercranial pressure (Eigsti and Henke, 2006; ter Laan et al., 2013). This autoregulatory mechanism in the core (cerebral) vasculature is widely different from systemic autoregulation and has been quantitatively investigated in various ways. The methods commonly used to study SNA include plasma kinetic analysis of the sympathetic neurotransmitters—norepinephrine and epinephrine (Reed et al., 1989; Esler and Kaye, 2000; Guild et al., 2010; Seravalle et al., 2013), direct recordings of neural activity from intrafascicular electrodes implanted in muscles (microneurography) (Anderson et al., 1989; Ramchandra et al., 2008; Van Vliet et al., 2008; Hart et al., 2017), heart rate variability analysis of electrocardiograms (ECG) (Appelhans and Luecken, 2006; Thayer et al., 2012; Deng et al., 2018; Hodges et al., 2018), low frequency analysis of impedance cardiography (Burgess et al., 2004), and analysis of the low-frequency oscillation in Photoplethysmograms (Nitzan et al., 1994, 2009; Penzel et al., 2002). However, these studies have been inconclusive, and there is a lack of PPG related studies investigating SNA. Whether or not sympathetic nervous activity (SNA) influences cerebral autoregulation in humans hence remains controversial (Hernandez et al., 1971; Ainslie and Brassard, 2014).

In addition to differential increases in sympathetic constrictor activity across the body during body cooling, local skin cooling (by immersion of limbs in ice water) is thought to amplify the direct constriction of the cutaneous vessels. During local skin cooling, the AVA constriction is prolonged, and with more exaggerated cooling (ice water immersion) there is sustained closure of AVAs. Hence, most studies evaluating autonomic function during cold employ one of these two methods of cold exposure [cold pressor test (CPT) or whole body cooling] (Silverthorn and Michael, 2013).

The primary focus of this paper is to understand and quantitatively assess the differences in degree of sympathetic control on peripheral and core vasculature during both local (CPT) and whole body cold exposure, using photoplethysmography (PPG). PPG is a non-invasive optoelectronic technique used to measure blood volume changes in the tissue vasculature by shining light into the tissue and detecting the light reflected or transmitted through the tissue during the cardiac cycle (Kyriacou et al., 2019). PPG is used since the vascular changes caused due to sympathetic and parasympathetic activation i.e., vasoconstriction or dilation will be directly reflected in the volumetric changes of the PPG signals. Both the investigations described in the paper utilize infrared PPG signals acquired from the ear canal (EC) and the index finger. The ear canal being supplied by the branches of the external carotid artery (superficial temporal artery and internal maxillary artery) which supply blood to face and neck represent the core circulation appropriately. The EC is also provide stable temperature close to the core temperature of the body (Shelley et al., 2006; Budidha and Kyriacou, 2014b, 2018; Venema et al., 2014). The finger, on the other hand, represents the peripheral circulation. The quantitative assessment of the differences in degree of SNA on the periphery and core was performed by measuring multi-site differential Pulse Transit Time (PTT)s during CPT. During prolonged whole-body exposure, this was performed through low-frequency analysis of the central and peripheral PPG signals.

## 2. Materials and Methods

### 2.1. Study I—Cold Pressor Test (CPT)

Twelve healthy volunteers (Seven male and five female with mean age ± SD: 26.2 ± 4.8 years) with no history of cardiovascular disease were recruited for a cold pressor test. This study was carried out in accordance with the recommendations of the Belmont report and the Research Council UK guidelines for Governance on good research conduct. The protocol of the study was approved by the City, University of London Senate Research Ethics Committee. The experimental protocol was clearly explained to all the participants and both written and informed consent was obtained prior to the experiment in accordance with the Declaration of Helsinki.

#### 2.1.1. Measurement Setup

The measurement setup involved the acquisition of PPG, electrocardiogram (ECG), and skin temperature signals. PPG signals were recorded from the right ear canal, left index finger (LIF), and the right index finger (LIF) of the volunteers. PPG signals from the ear canal were recorded using a previously developed earphone shaped PPG sensor (Budidha and Kyriacou, 2014b). The peak emission wavelength of the infrared LED in the sensor was 870 nm. The photodetector used was a flattop photodiode with an active area of 0.65 mm^2^ and peak sensitivity at 900 nm. Two reflectance finger PPG probes, optically identical to the ear canal probe were also developed to facilitate the acquisition of PPGs from the right and left index fingers. Two thermistor based fast response temperature transducers were used to measure the skin temperature of both hands. PPG, ECG, and temperature signals were acquired using a research PPG system (ZenPPG) previously developed by the Research Centre for Biomedical Engineering (RCBE) at City (Budidha et al., 2018). ZenPPG is a modular, dual-wavelength and dual channel research PPG acquisition system. The main functions of the device are the interface with custom-made and/or commercial PPG/SpO_2_ sensors, the intermittent switching of light sources, the sampling of red/infrared signals, and the output of the signals to data acquisition systems. The data acquisition card used was a USB-6212 mass termination DAQ card from National Instruments (National Instruments Inc. Austin, TX, USA). A detailed description of the instrumentation unit and the relevant hardware is presented in Budidha and Kyriacou (2014b, 2018) and Budidha et al. (2018).

#### 2.1.2. Experimental Protocol

The experiments were carried out at the RCBE laboratory, City, University of London, under room temperatures between 24 ± 2°C. During the experiments, the subjects were made to sit on a comfortable chair, with both hands resting on soft cushions, arranged to a height equivalent to their heart position. The subjects were asked not to smoke or exercise for at least two hours before the experiment. All volunteers were rested for approximately 10–15 min before the start of the study to acclimatize to room temperature (Nitzan et al., 1994; Korhonen, 2006). The volunteers were asked to keep their movement to a minimum during the study to avoid motion artifacts on the acquired signals. Once the volunteer was comfortable in the position, the ear canal, and the finger PPG sensors were connected to the respective locations; the skin temperature sensors were attached to the dorsal surface of the right and left hand of the volunteer; the Ag-AgCl easitab ECG electrodes (SKINTACT, F-WA00) were attached to the volunteer in the chest (lead-I configuration).

Once all the sensors were in place, baseline readings were obtained from all the volunteers for 2 min, before they were notified with a countdown (3–1) to start slowly immersing their right hand into the ice bath maintained at approximately 1°C. After thirty seconds into the ice bath, they were notified again to start slowly removing their right hand from the ice bath and place it back on the surface. The monitoring was continued until the volunteer's hand rewarmed to a minimum temperature above 24°C. The minimum and maximum time it took the volunteers to rewarm their hand to 24°C was 7 min 9 s and 12 min 30 s. More information regarding the protocol is presented in Budidha and Kyriacou (2014a,b).

#### 2.1.3. Data Analysis

**Figure 1:**
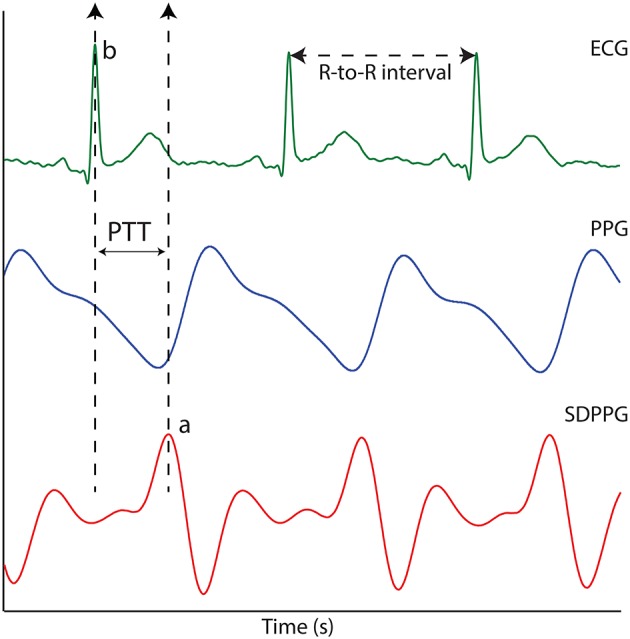
The ECG signal, the PPG signal and the second derivative photoplethysmographic (SDPPG). PTT is the time difference between points “a” and “b”.

The raw PPG, ECG, and temperature data recorded during the study were extracted separately for offline analysis. Prior to any signal processing routines, all the acquired signals were resampled from the acquired sampling rate (1,000 to 100 Hz). This was to eliminate any unwanted noise and to condition the acquired signals. The PPG signals were filtered using an equiripple band-pass filter with cut-off frequencies 0.4 and 10 Hz. The ECG signal was filtered using an equiripple low pass filter with a cut-off frequency of 40 Hz. The PTT was calculated by using the foot of the PPG and the R-wave peak of the ECG signal. The foot of the PPG signal was detected using the second derivative maximum method. This was to increase the accuracy and detection rate of the inflecion points. The second derivative of the infrared PPG signals was acquired using the SavitzkyGolay method with a window width of 31 points. The maximum of the second derivative PPG signal (SDPPG) represented the maximal acceleration point at the foot of the PPG signal. The time difference between the R-wave peak of the ECG signal and the maximum peak of the SDPPG was then used to calculate PTT as shown in Figure 1. Using the SDPPG signals from the EC, the LIF, and the RIF, the mean PTT was calculated for all three stages of the experiment (before, during, and after the ice immersion) for each volunteer. The mean PTT calculated from the EC, the LIF, and the RIF were then compared during the CPT.

To check if there were any statistically significant differences between the PTT measured during baseline, ice water immersion, and the recovery period of the experiment in all three locations, statistical analysis was performed using SigmaPlot 12.0 (Systat Software Inc., Chicago, USA). Prior to the statistical tests, the normality of the data was tested using the Kolmogorov–Smirnov test. However, due to the small sample size of the experiments, a non-parametric test was used to check if mean PTT of the EC, the RIF, and the LIF were significantly different during the three stages of the experiment. The test used was Kruskal–Wallis One Way Analysis of Variance on Ranks. The parametric equivalent of the Kruskal–Wallis test is the one-way analysis of variance (ANOVA). A comparison of two groups of data was recognized as significantly different if the computed *P*-value by Kruskal–Wallis test was < 0.05.

### 2.2. Study II—Whole Body Cooling

Twelve healthy volunteers (six-female and six-male) aged between 19 and 45 (mean age ± SD—28 ± 5 years) were recruited for this study up on approval from City, Senate Research Ethics Committee. Based on the medical history and a general medical examination, subjects with cardiovascular, pulmonary, or metabolic diseases were excluded from the study. The general medical examination included a measure of heart rate, blood pressure, and core body temperature. All the subjects were normotensive (mean BP ± SD: 116/70 ± 14/11 mmHg), normothermic (mean core temp ± SD: 36.52 ± 0.33^*o*^C ) and none was taking any medication. The subjects were informed of the details of the study and a signed informed consent was sought from all the volunteers before the experiment. Similar to the CPT, all the subjects were asked to refrain from ingesting beverages containing caffeine and alcohol and were asked not to exercise or smoke for at least two hours preceding the test. To maximize the effect of cold temperatures on the cardiovascular system, all the subjects were asked to wear just one layer of clothing during the experiment.

#### 2.2.1. Measurement Setup

Similar to the CPT study, the measurement setup involved the acquisition of PPG signals from the ear canal and the left index finger, ECG signal, and temperature measurements from the periphery (skin) and the core (tympanic). PPG and ECG measurements were made using the ZenPPG system. Core temperature was measured using a tympanic thermometer (ThermoScan-5 IRT4520, Braun GmbH, Frankfurt, Germany). Skin temperature was measured using the laser Doppler flowmeter (LDF) (moorVMS-LDF2, Moor Instruments, Devon, UK).

#### 2.2.2. Measurement Protocol

The trials were carried out in the RCBE laboratory at City. Upon arrival, all the volunteers were seated in a room maintained at 24±1°C for a minimum of 10 min to ensure hemodynamic stabilization. During the study, the subjects were made to sit in a comfortable chair, with both hands resting on the armrests arranged to a height approximately equivalent to their heart's position. Once the volunteer was comfortable, heart rate (HR), blood pressure, and core temperature were measured. If the volunteer was found to be normotensive and normothermic then the study was continued. The finger and ear canal PPG probes were then attached to the left index finger and the left ear canal of the volunteer, respectively. The skin temperature sensor from the LDF was placed just below the thumb on the dorsal surface of the left hand and it was attached to the skin by means of a ring-shaped double-sided adhesive. Finally, the ECG cable was connected to the Ag-AgCl easitab ECG electrodes (SKINTACT, F-WA00) placed directly on the chest (the right and the left side) and the left hip.

Once all the sensors were in place, the investigation protocol started with the acquisition of baseline measurements from the volunteer for at least 2 min. The volunteers were then moved to the adjacent temperature-controlled room maintained at 10±1°C for 10 min. After the cold exposure, the volunteers were moved back to normal room temperatures (24°C), where monitoring continued for another 10 min. PPG, ECG, and temperature data was continuously recorded during all three phases of the experiment. The core temperature was measured from the right ear of the volunteer once every minute for the entire duration of the study (22 min). Blood pressure was measured once at the start of the study, and then at the end of the cold exposure and the recovery period. More information regarding the protocol is presented in Budidha and Kyriacou (2018).

#### 2.2.3. Data Analysis

The infrared PPG signal acquired from the finger and the ear canal was imported and preprocessed for analysis in MATLAB *(The Math Works Inc., Massachusetts, USA)*. The acquired signals were bandpass filtered, with a linear-phase FIR filter. The lower and upper cut-off frequencies of the filter were 0.035 and 0.5 Hz, respectively. The signals were filtered to eliminate all other components apart from the sympathetic and the parasympathetic components, which are known to be between 0.04 and 0.4Hz (Billman, 2011). The filtered signals were then divided into three segments, each containing information from one stage of the study, i.e., the baseline, the cold exposure, and the recovery. The power spectrum of all three segments was then obtained using the “pwelch” function in Matlab. From the power spectrum, the power of the low-frequency components (LF) (between 0.04 and 0.15 Hz) and the high-frequency (HF) components (in the range 0.15–0.4 Hz) was computed. The power of LF components corresponds to both sympathetic and parasympathetic activity while the power of HF components corresponds to only parasympathetic activation. Hence, the ratio of LF to HF (LF/HF) components was calculated to quantify the changes in the sympathetic activity, as in Pagani et al. (1984). The LF to HF ratio measured from the finger and the ear canal were then compared during all three stages of the experiment. Kruskal–Wallis One Way Analysis of Variance on Ranks was performed on the data to check if the changes occurring in the LF/HP ratio are significantly different between any of the two stages in the study. Before the statistical analysis, the normality of the data was tested using the Kolmogorov–Smirnov test with Lilliefors' correction. A comparison between data acquired from two different stages of the experiment was recognized as significantly different if the computed *P*-value by Kruskal–Wallis test was <0.05. A detailed description of the analysis is presented in Budidha (2016).

## 3. Results

### 3.1. Pulse Transit Time (PTT)

**Figure 2:**
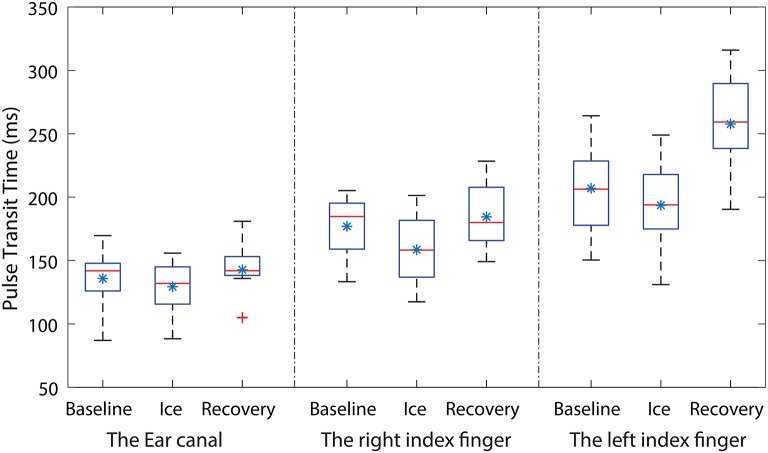
Box-plots showing the changes in PTT measured during all three stages of the experiment from the ear canal (EC), the right index finger (RIF) and the left index finger (LIF). Each box represents the interquartile range of the mean PTT measured from 12 volunteers. The red line in each box shows the median value of the data, the ^*^ shows the mean and + shows the outliers.

PPG signals with high quality were acquired from the EC, the RIF, and the LIF of all the volunteers, during all phases of the experiment. The mean PTT measured from the EC, the RIF, and the LIF PPG signals of each volunteer during all three stages of the experiment is graphically shown using a box-plot in Figure 2.

From Figure 2, the mean baseline PTT measured from the EC is smaller than the PTT measured from the LIF and the RIF. The mean PTT measured from the EC, the RIF, and the LIF has dropped during the ice water immersion. However, the percentage drop in PTT varied across all three locations. The PTT in the EC and the LIF has dropped by 5 and 7% while the RIF PTT has dropped significantly by 12%. The skin temperature of the RIF during ice immersion decreased to 8.1 ± 0.936 °C from 29.3 ± 0.9 °C during baseline, while the LIF skin temperature did not change. During the recovery period, the mean PTT measured from the EC has returned to the baseline value, whereas the RIF and the LIF PTT exceeded baseline values. The percentage increase in mean PTT during the recovery period, when compared to baseline, was 4% in the RIF and 20% in the LIF. These observations demonstrate the ear canal is much more resistant to autonomic activity (local changes in BP) compared to the peripheral sites.

To check if there were any statistically significant differences between the PTT measured during baseline, ice water immersion and the recovery period of the experiment in all three locations, statistical analysis was performed on the measured data. All the data was found to be normally distributed. A significant difference was found between the groups. To isolate the group (or groups) that differ from the others, Dunnett's Method (Multiple Comparisons vs. Control Group) was used. The baseline PTT from each location was considered the control group. The results of the statistical tests together with the corresponding H value are shown in Table 1.

**Table 1:** Summary of the statistical test results obtained from the Kruskal–Wallis test performed on the PTT measured from EC, RIF, and LIF of the volunteers.

**Location**	**Comparison**	***P* < 0.05**	***H*-value**
Ear canal	Baseline vs. Cold	No	2.2
	Baseline vs. Recovery	No	
Right index finger	Baseline vs. Cold	Yes	4.8
	Baseline vs. Recovery	No	
Left index finger	Baseline vs. Cold	No	12.3
	Baseline vs. Recovery	Yes	

*A P-value < 0.05 indicates a statistically significant difference. The highest H-value corresponds to the largest discrepancy between rank sums*.

From Table 1, a statistically significant difference in PTT was found in RIF during ice immersion and in LIF during recovery. No statistically significant changes were found in the ear canal during any phases of the experiment.

### 3.2. Whole Body Cooling

**Figure 3:**
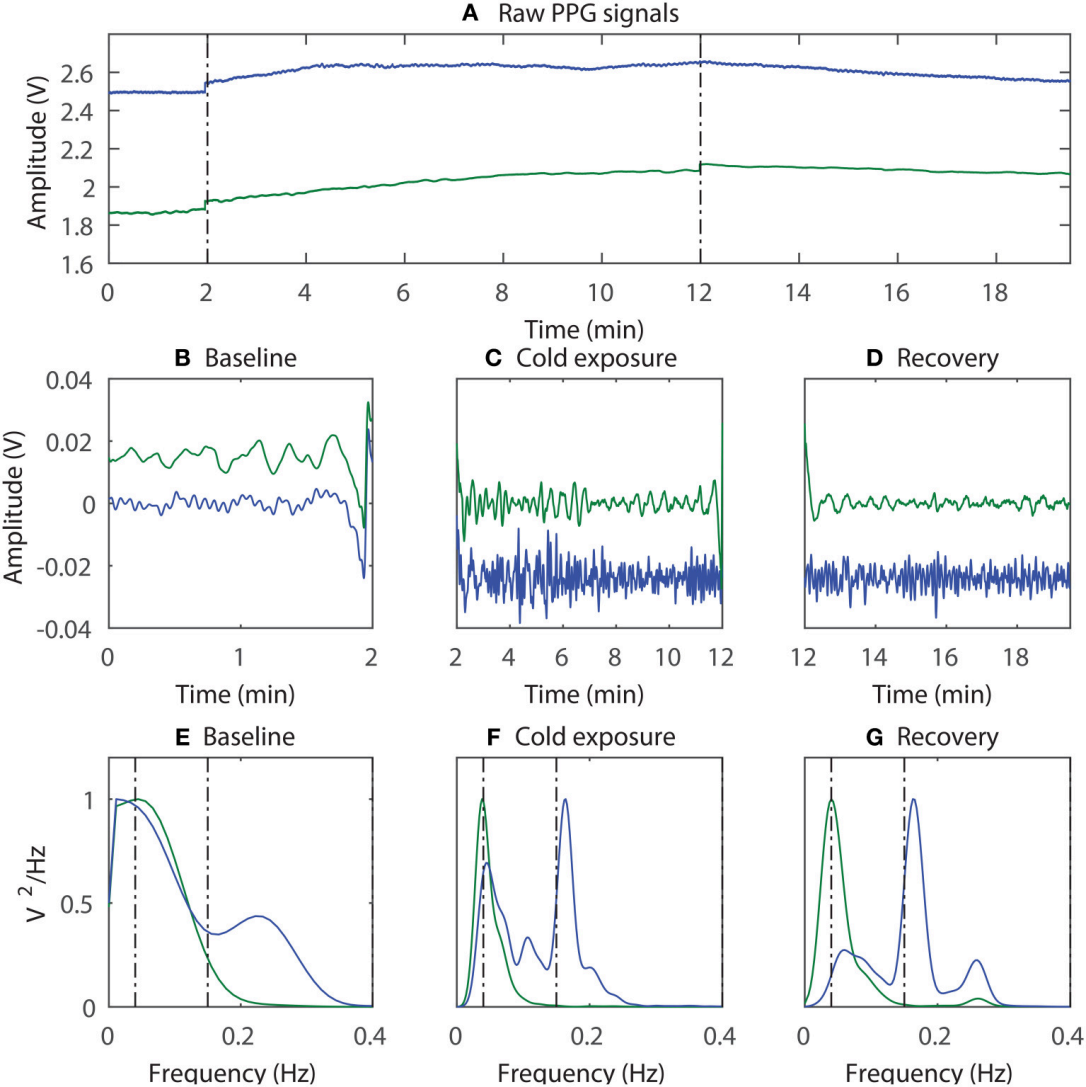
An example of the sympathetic activity estimation; **(A)** The raw PPG signals acquired from the ear canal (blue trace) and the finger (green trace) were first bandpass filtered and then divided into three segments, each containing data from **(B)** baseline, **(C)** cold exposure, and **(D)** recovery; The power spectrum of the corresponding signals was then acquired **(E–G)**.

To quantify the differences in the degree of SNA between the periphery and the core during prolonged whole body cooling, low-frequency spectral analysis of the PPG signals was performed. The power spectrum of the ear canal (blue trace) and the finger (green trace) PPG signals acquired from a volunteer during (Figure 3E) baseline, (Figure 3F) cold exposure, and (Figure 3G) recovery periods is shown in Figure 3, along with (Figure 3A) the raw and (b,c and d) the filtered PPG signals. Note that the power spectrum acquired at each stage was normalized to the component with the maximum power to facilitate easy comparison between the two locations. Therefore, the power of the highest component in each sub-plot is at 1.

**Figure 4:**
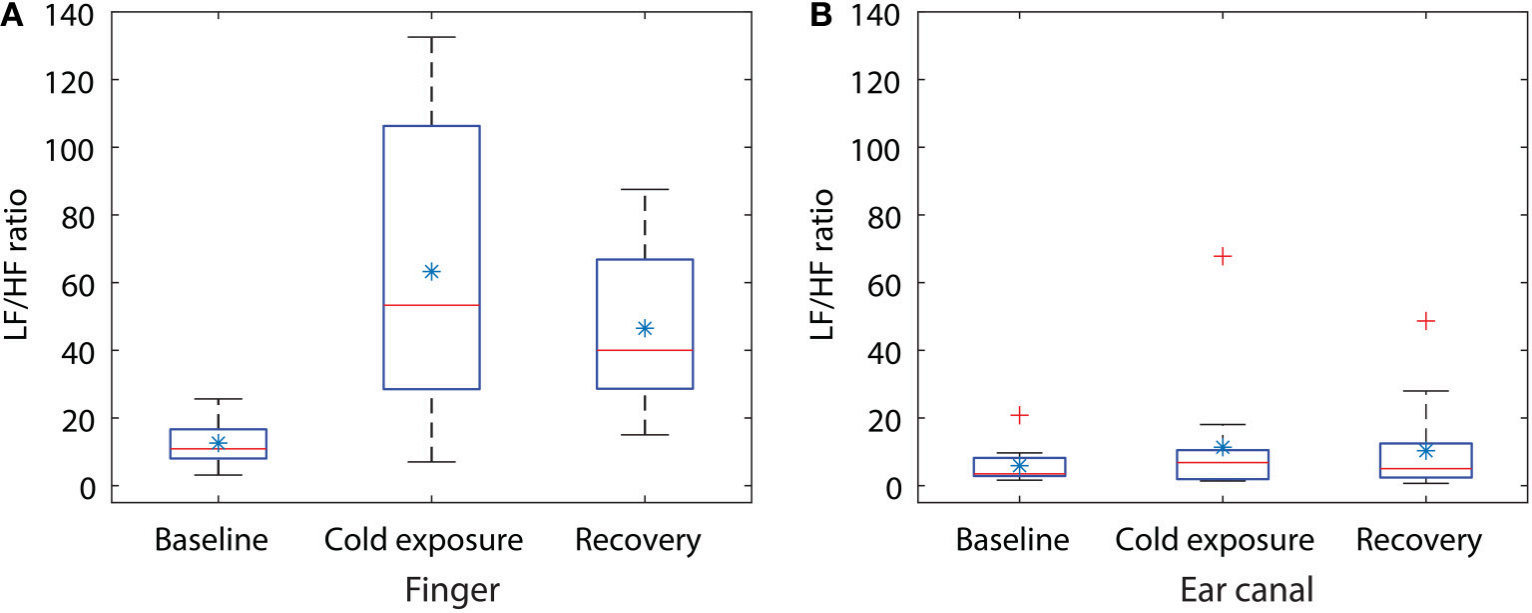
Box plot showing the changes in LF/HF ratio during the cold exposure in **(A)** finger and **(B)** the ear canal. Each box represents the interquartile range of the mean LF/HF power ratio measured from 12 volunteers. The red line in each box shows the median value of the data, the ^*^ shows the mean and + shows the outliers.

From Figure 3, the component with the maximum peak in both the finger and the ear canal during baseline was the LF component. This indicates the presence of sympathetic and parasympathetic activity, which is expected for normal homoeostasis. During the cold exposure (Figure 3F), the component with the maximum peak in the finger was the LF component, whereas in the ear canal it was the HF component. Hence, the ratio of LF to HF will be very high in the finger compared to the ear canal, suggesting a higher degree of SNA in the periphery. The LF/HF ratio of the finger has increased to 100.08 during the cold exposure from 16.86 at the baseline. While the ear canal LF/HF ratio has decreased to 1.43 during the cold exposure from 1.91 at baseline. These results indicate the differences in the degree of SNA between the two locations. During recovery (Figure 3G), the power of the LF component in finger PPG signals is reduced compared to cold exposure. Hence, the ratio of the LF/HF power in the finger will be decreased, implying the increase in parasympathetic activity. The LF/HF power ratio estimated from the finger and the ear canal PPG signals during baseline, cold exposure and recovery in all 12 volunteers is graphically displayed in Figure 4.

Statistical tests were performed on the data to check if the changes occurring in the LF/HP ratio are significantly different between any of the two stages in the study. Some of the data included in the study were not normally distributed, hence a non-parametric test (Kruskal–Wallis One Way Analysis of Variance on Ranks) was used for statistical analysis. Similar to CPT study, the groups with statistical significance were isolated from other groups using the Dunnett's Method, where the baseline LF/HF power ratio was as a control comparison group. The results of the statistical tests together with the corresponding P-value are shown in Table 2. A significant difference was found when the LF/HF power ratio computed from the finger was compared between baseline and the cold exposure, while no significant differences were found in the ear canal LF/HF ratio in any of the comparisons. A statistically significant difference was also found in LF/HF power ratio when baseline reading was compared with recovery. This indicates that the volunteers have not entirely recovered from the cold exposure in the monitoring period. These results prove that the sympathetic control of the blood vessels in the periphery is significantly higher than the core regions and that these differences can be calculated quantitatively using PPG.

**Table 2:** Statistical test results obtained from the non-parametric analysis performed on the LF/HF ratio measured from the finger and the ear canal PPGs.

**Location**	**Comparison**	***P* < 0.05**	***H*-value**
Finger	Baseline vs. Cold	Yes	19.12
	Baseline vs. Recovery	Yes	
Ear canal	Baseline vs. Cold	No	0.115
	Baseline vs. Recovery	No	

*A P-value < 0.05 indicates a statistically significant difference. The highest H-value corresponds to the largest discrepancy between rank sums*.

## 4. Discussion

The degree of sympathetic control over cutaneous blood vessels when exposed to cold is thought to be more prominent in the periphery than the core vasculature but still remains controversial. Though cerebral autoregulation has been reported to be preserved in sympathetically and parasympathetically denervated animals (cats), which suggest that SNA does not have a major role in cerebral autoregulation (Busija and Heistad, 1984). This paper aims to investigate the possibility of using photoplethysmography as a tool to quantitatively differentiate the effect of SNA on peripheral and core blood vessels. Two methods utilizing PPG were proposed, namely differential multi-site PTT measurements and low-frequency spectral analysis to quantitatively determine the degree of SNA. The former was used during short-term local cold exposure (CPT) and the later in long-term whole body cold exposure. PPG signals were acquired from the ear canal and the finger as they closely represent the cerebral (core) and peripheral circulation, respectively (Budidha and Kyriacou, 2014b, 2018).

The rationale for using these two methods was that exposure to cold stress causes the activation of sympathetic nerves intervened within the heart and blood vessels. This, in turn, causes an increase in heart rate and a decrease in the blood vessel diameter. These reactions combine to an increase in the velocity of the pulse propagating through arteries and the blood pressure. Conversely, after the cold exposure, the dilation of blood vessels to rewarm the body causes a decrease in pulse wave velocity (PWV) and blood pressure (BP). The velocity of a pulse propagating through the arteries is given as the pulse propagation distance by the Pulse Transit Time (PTT) (Smith et al., 1999). Therefore, PTT is indirectly proportional to the PWV and the BP. By calculating PTTs from the PPG signals acquired from two or more sites, and monitoring the changes in the measured time during all three phases of the experiment, the extent of vasoconstriction and local change in PWV at a given site can be indirectly measured (Penzel et al., 2002; Budidha and Kyriacou, 2014a). Similarly, the extent of vasodilation after the cold exposure can be demonstrated indirectly.

The frequency at which the sympathetic and parasympathetic nerves control the heart's activity is known to be between 0.04 and 0.15 Hz (LF) and 0.15–0.4 Hz (HF), respectively. These spontaneous oscillations in the heart rate and blood vessel diameter are reflected in the PPG signal as a change in time between two consecutive pulses and as a change in PPG signal amplitude respectively. Hence, by measuring the heart rate variability from PPG signals or by directly performing a power spectrum analysis on raw PPG signals acquired during cold exposure, the power of the sympathetic and parasympathetic activity can be directly determined. However, SNA driven oscillations were found to be more pronounced in the power spectrum analysis on raw PPG signals than the frequency domain analysis of heart rate variability (Nitzan et al., 1994, 1998, 2009). Hence, the degree of sympathetic control over blood vessels in the finger and the ear canal was studied through low-frequency spectral analysis of the PPG signals acquired from the respective areas.

During the CPT, multisite PTT measurements were made using PPG signals acquired from the LIF, the RIF, and the EC. The PTT measured from the EC during baseline measurements was smaller than the RIF and LIF. This is expected since the ear is closer to the heart than the finger. During the ice water immersion, the amplitude of the PPG signals acquired from the periphery (RIF, LIF) has reduced as soon as the right hand was immersed into the ice bath. Similarly, as anticipated, there was a reduction in the PTT measured from the RIF (12%) and the LIF (7%) during ice immersion. This indicates a generic raise in SNA and systemic vasoconstriction from the exposure of the periphery to cold. This indirectly also indicates the general rise in PWV and BP in the body. From these observations, it will be a fair assessment to suggest that multisite PTT measurements can detect autonomic arousal with a minimal effort (Penzel et al., 2002). The reduction in PTT, however, was statistically significant (*P*<0.05) only in the RIF (Table 1). The disparity in the strength of local vasoconstriction in the RIF is expected since sympathetic activation will be more prominent in the right hand which is immersed in ice water than other locations. During recovery, the PTT measured from RIF has exceeded the baseline due to the vasodilatory effects of the parasympathetic system. However, this effect was not statistically significant within the monitoring period. The LIF has shown a significant difference in PTT measured during the recovery period, indicating profound vasodilation to rewarm the periphery. The ear canal being supplied by the core circulation has shown no significant changes in PTT during ice immersion (5%), and during recovery has attained a value close to the baseline. From these observations it is clear that profound systemic (peripheral) vasoconstriction can result from exposure of a peripheral limb to cold, however, these systemic changes do not influence the core circulation significantly.

During whole-body cold exposure, the frequency spectrum of the infrared finger and the ear canal PPG signals were acquired, and the power of the LF and HF component was calculated. The mean LF/HF power ratio of the finger was larger than the EC during baseline reading. The mean LF/HF power ratio of the finger raised from 12.5 to 63.27 (80% raise) during cold exposure, while the ear canal LF/HF power ratio raised from 5.91 to 11.35 (48% raise). The LF/HF ratio in the finger was found to be statistically significant (*P* = 0.002) during the cold exposure. The ear canal LF/HF ratio, on the other hand, remained relatively constant (*P* = 0.781). Similar results were reported by Tansey et al. when temperature changes in the periphery and core were used as a marker for thermoregulation and autonomic control of blood flow (Tansey et al., 2014). During the recovery period, the LF/HF power ratio of the finger and the ear canal have reduced down to 46.5 and 10.3, respectively. This indicates that the volunteers have not fully recovered from the effects of cold within the monitoring period. The difference between the rise in LF/HF ratio signifies the disparity in the degree of SNA between the peripheral and core circulation. These results are in agreement with the view that, neurogenic control has little influence on cerebral or core autoregulation. These finding are also in agreement with the results of power spectrum analysis of PPG signals by Nitzan et al. (1994).

Although both methods produce reliable results in studying the degree of sympathetic/parasympathetic activity, there is one key disadvantage. The PTT based measure can only be used in short-term cold exposure studies (such as CPT), as prolonged exposure to cold causes, the amplitude of the PPG signal reduce significantly to a point where the heart pulses are indistinguishable from noise. This will result in inaccurate detection of PPG signal peaks and hence an inaccurate estimation of PTT. Similarly, the low-frequency spectral analysis of the PPG signal can also be used only in long-term cold exposure studies as during short exposure to cold, such as in CPT, the data acquired will not be long enough to contain the sympathetic and parasympathetic frequencies. Nevertheless, both the methods proposed in this paper have comprehensively demonstrated their use in estimating the degree of sympathetic activity.

Compared to the conventional methods of measuring the degree of sympathetic activity such as plasma kinetic analysis of the norepinephrine, microneurography, or impedance cardiography, the proposed methods possess several advantages. Firstly, both the proposed methods are non-invasive and are not as time-consuming and demanding for both the experimenter and the subject as the conventional methods. The measurement of neurotransmitter levels in the blood is not a true indicator for the degree of total body SNA as only a small fraction (5–10%) of the amount of norepinephrine secreted from nerve terminals is circulating in blood (Sinski et al., 2006). The single unit recording in microneurography limits the analysis to the one nerve fiber, whereas the features and the amount of activity in parallel neural elements remain unknown (Vallbo et al., 2004). The accuracy of impedance cardiography is also very limited in assessing cardiac function as the equations used to calculate the volumetric estimates are based on simplified physiological assumption (Burgess et al., 2004). The proposed PPG based methods, however, do also have pitfalls compared to conventional methods. Similar to the HRV analysis, the multi-site PTT and power spectrum analysis of PPGs are influenced by factors such as body position, physical, and mental activity, body mass index, sex, or even diet. Also, elevated indices (e.g., LF/HF ratio) are not direct representation of tonic activity, but rather the result of their influence on the effectors. Finally, all these methods including the ones proposed in this paper does not allow large movements of the subject, which is a limitation particularly in studies of the motor systems (Vallbo et al., 2004). Nonetheless, the proposed methods have shown promise and can be used in clinical environments such as in patients undergoing major surgery (coronary artery bypass surgery or gastric bypass surgery), or in healthy blood donors for assessing their sympathetic tone. Especially, the methods can be used in identification of patients at high risk of heart failure and in assessing the impact of pharmacological and non-pharmacological interventions within individual patients. Other areas in which the methods might prove useful are in studying the characteristic changes in autonomic nerve functions due to sleep disorders such as sleep apnoea and stress studies.

In conclusion, differential multi-site PTT measurements using PPGs and/or low-frequency spectral analysis of PPG signals can be used to quantitatively estimate the intensity of sympathetic activity during cold stress. The results show the core/cerebral circulation to be preserved during local or whole body cold exposure when compared to the periphery. Further evaluation in a large group of volunteers or critically ill patients will, however, be required to further asses the credibility of the methods.

## Author Contributions

KB together with PK has identified the gaps in the literature and formed a hypothesis for using PPG as a tool for quantitative analysis. KB hired the volunteers, carried out the experiments, collected the data, and carried out the relevant data analysis as described in the manuscript. KB and PK have both participated in writing this manuscript.

### Conflict of Interest Statement

The authors declare that the research was conducted in the absence of any commercial or financial relationships that could be construed as a potential conflict of interest.
